# Electrically-controlled suppression of Rayleigh backscattering in an integrated photonic circuit

**DOI:** 10.1515/nanoph-2023-0431

**Published:** 2024-01-08

**Authors:** Oğulcan E. Örsel, Jiho Noh, Gaurav Bahl

**Affiliations:** Department of Electrical & Computer Engineering, Urbana, USA; Department of Mechanical Science & Engineering, University of Illinois at Urbana-Champaign, Urbana, IL 61801, USA

**Keywords:** acousto-optics, time-reversal symmetry breaking, integrated photonics, chiral dispersion, backscattering, Rayleigh scattering

## Abstract

Undesirable light scattering is a fundamental cause for photon loss in nanophotonics. Rayleigh backscattering can be particularly difficult to avoid in wave-guiding systems and arises from both material defects and geometric defects at the subwavelength scale. It has recently been shown that systems exhibiting chiral dispersion due to broken time-reversal symmetry (TRS) can naturally mitigate Rayleigh backscattering, yet this has never been explored in integrated photonics. Here we demonstrate the dynamic suppression of disorder-induced Rayleigh backscattering in integrated photonics even when defects are clearly present. Our experiments are performed using lithium niobate on insulator resonators in which TRS is broken through an electrically-driven acousto-optic interaction. We experimentally observe near-complete suppression of Rayleigh backscattering within the resonator by measuring the optical states and through direct measurements of the back-scattered light. We additionally provide a new and intuitive generalization argument that explains this suppression of backscattering as a form of topological protection in synthetic space.

## 1 Introduction

**Figure 1:**
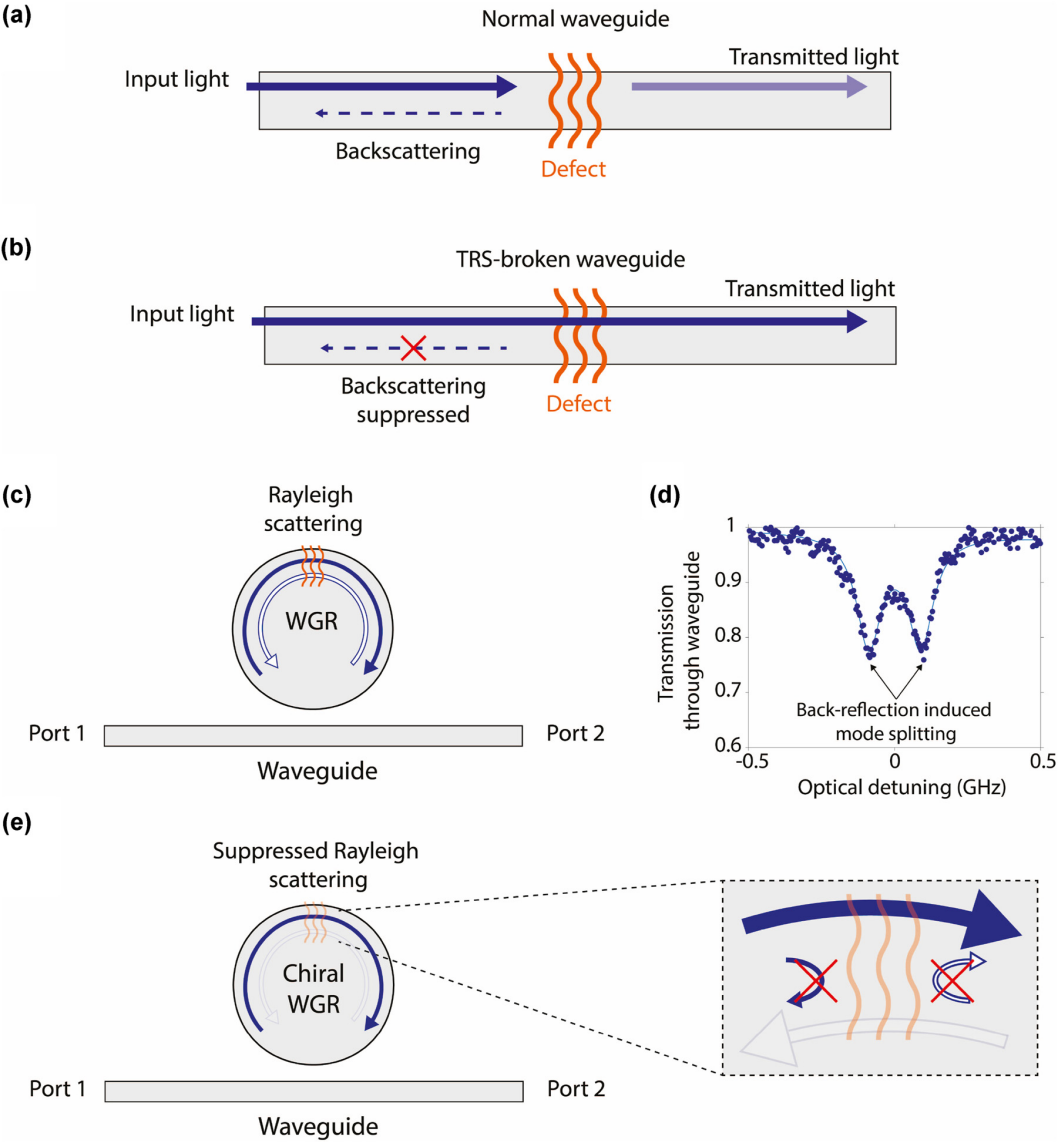
Backscattering suppression via chiral dispersion engineering. (a) A 1D waveguide supports symmetric forward and backward propagating optical modes. Subwavelength defects can produce undesirable Rayleigh backscattering that reduces forward transmission. (b) In a waveguide demonstrating a chiral dispersion through broken time-reversal symmetry the back-propagating states are not available, which suppresses the backscattering from taking place in spite of the presence of the defect. (c) Similarly, whispering gallery resonators (WGRs) are quasi-1D systems that support nominally degenerate cw and ccw optical modes. Rayleigh backscattering within the resonator can couple these counter-propagating modes leading to the loss of their distinguishable directionality. (d) Experimental example of a doublet of cw/ccw hybridized modes induced by Rayleigh scattering in an integrated LNOI WGR shown in later figures. (e) In a chiral WGR, the optical density of states for cw and ccw circulation can be different, which as we show can suppress the Rayleigh backscattering.

Defects are unavoidable in almost every optical device [1], [2], and especially in integrated photonics [3], [4], [5] due to the limitations of crystal growth and nanofabrication methods. Common subwavelength defects like intrinsic material stresses, density variation, and surface roughness can lead to Rayleigh scattering which can result in extra propagation loss, limitations on optical Q-factors, and undesirable inter-modal conversion [6], [7], [8]. In particular, disorder-induced Rayleigh backscattering (Figure 1a) is a common problem in waveguiding structures that has been directly linked to stability problems in frequency combs [9], [10], elimination of directional gain in micro-ring lasers [11], and increased bit error rates in integrated modulators [12]. While devices like isolators and circulators serve to block undesired back-scattered light, they do not address the fundamental issue of back-scattering itself. Consequently, even though these devices offer some protection the photon directionality is compromised, leading to diminished power transfer and, in many cases, coupling into undesirable modes.

One approach to prevent disorder-induced backscattering is to strive for a perfect disorderless optical system, that is, by reducing or eliminating scatterers from the medium. However, since no fabrication method can produce truly defect-free optics, and sometimes defects can be introduced during operation or appear dynamically due to thermal fluctuations, this approach is not entirely feasible. A better alternative is to induce a chiral dispersion within the optical wave-guiding system so that only the mode in the desired propagation direction is supported while the counter-propagating mode (which would otherwise have received the backscattered light) is either eliminated or displaced in frequency-momentum space. This results in a large contrast in the optical density of states (ODoS) and inhibits the backscattering from taking place at all. Such chiral ODoS can be achieved by breaking time reversal symmetry (TRS) within the medium (Figure 1b), by specifically engineering a reduced spectral overlap for the scattering pathway. TRS can be naturally broken in magneto-optic materials and the inhibition of coherent backscattering from nanoscale defects has indeed been experimentally observed once previously in bulk crystals [13]. Unfortunately, this is not a convenient option for integrated photonics due to the foundry-incompatibility of the materials and the need for magnetic biasing to break the TRS. Chiral dispersions are also possible in 2D fermionic systems, e.g., via the quantum Hall effect (QHE) [14]. Optical analogues of the QHE can also be realized in photonic topological insulator (PTI) metamaterials, using both gyromagnetic effects in photonic crystals [15], [16] and Floquet engineering [17], but these approaches are also not currently practical for integrated photonics. Importantly, topological protection in PTIs in general only applies to defects that do not violate the underlying protective symmetries and TRS-breaking is essential to protect against nanoscale defects [18]. There are indeed more practical approaches that are capable of breaking TRS and producing chiral dispersions, such as through optomechanics [19], [20], acousto-optics [21], [22], [23], [24], [25], [26], and electro-optics [27], [28], [29], which may be applied in photonic integrated circuits. However, as far as our knowledge extends, there have been no experiments exploring the suppression of disorder induced backscattering in integrated photonics.

## 2 Results and discussion

In this work, we experimentally demonstrate near-complete dynamic suppression of disorder-induced back-scattering within an integrated photonic system by means of electrically-driven acousto-optic interaction. For the demonstration of this effect we consider high Q-factor whispering gallery type resonators (WGRs), such as those with ring or racetrack geometry, in which backscattering can often be observed from deep subwavelength defects. A WGR is essentially a 1D wave-guiding system with periodic boundary conditions that supports counter-propagating optical modes that are frequency degenerate, nominally orthogonal, and form momentum-reversed pairs. Subwavelength scatterers can induce coupling between such counter-propagating mode pairs (see Figure 1c) causing the optical states to be disturbed from their intrinsic state in both directions. For a low backscattering rate the modes simply broaden from their intrinsic linewidth [7]. With higher backscattering rates (i.e., more than the loss rate of the optical mode) the modes exhibit distinct doublet characteristics as shown in Figure 1d, which is a clear signature of Rayleigh backscattering [1], [2], [3], [7], [30], [31]. If we can dynamically induce a strong chiral dispersion within this resonator by breaking the TRS (Figure 1e), the back-scattering should be suppressed, removing the doublet and restoring the original modes with an improved quality factor. We recently demonstrated a practical time-reversal symmetry breaking technique that can produce strong chiral dispersion within WGRs [21], [26]. In this work we employ this approach as a key enabler to investigate the distinct physics of back-scattering suppression via chiral dispersion engineering.

**Figure 2:**
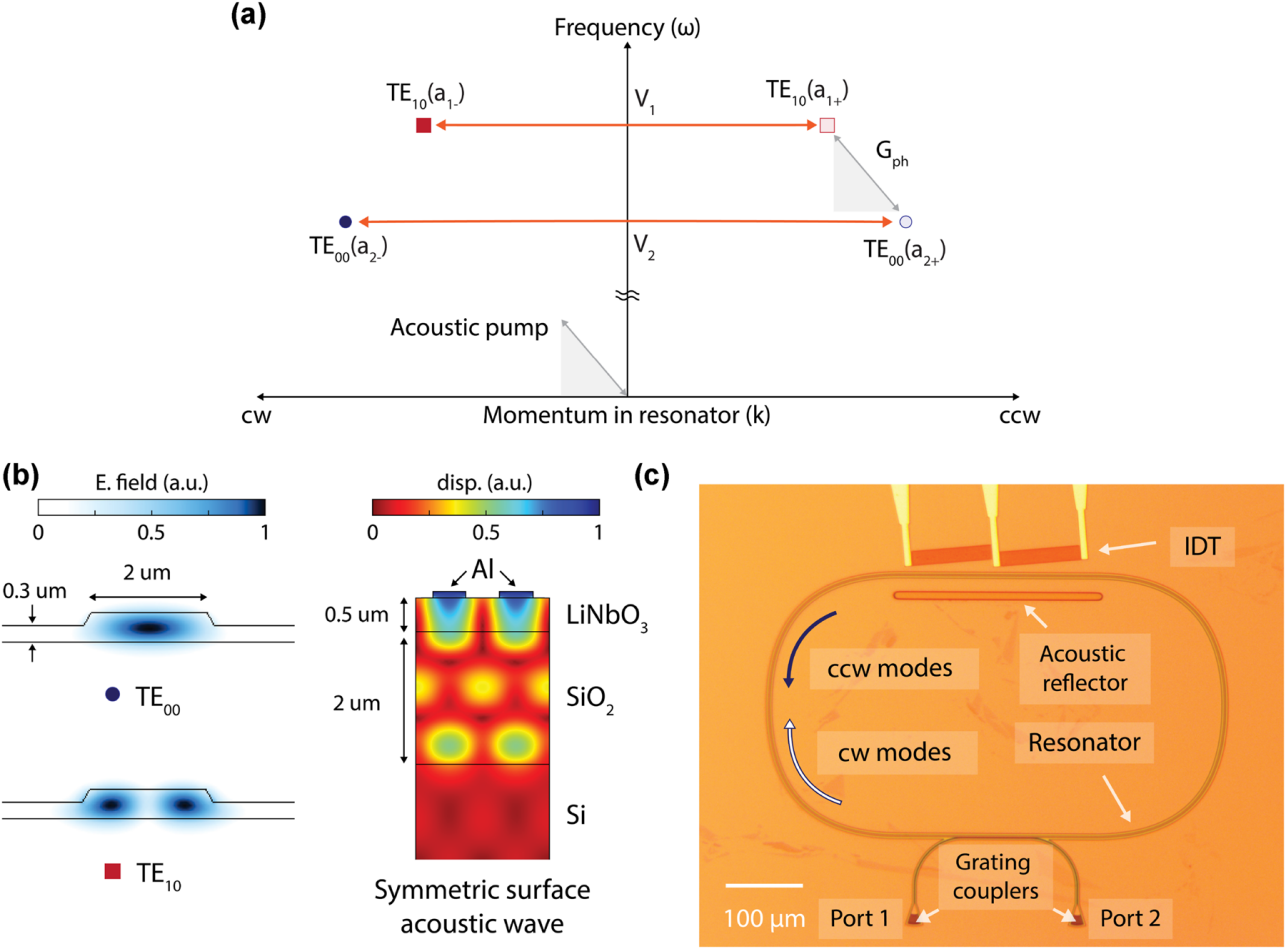
Implementation of chiral dispersion in LNOI racetrack resonators. (a) This frequency-momentum (*ω* − *k*) diagram shows the modes of the racetrack resonator in the presence of direction-sensitive acousto-optic interaction (*G*_*ph*_) and also disorder-induced backscattering (*V*_1_, *V*_2_). The relative placement of optical modes in *ω* − *k* space matches the experimental devices presented in this work. It can be seen that, when the acoustic pump has momentum in the cw direction, the phase matching for acousto-optic scattering is only satisfied for counterclockwise (ccw) circulation around the racetrack. (b) The resonator is designed to support TE_00_ and TE_10_ optical modes as simulated here (blue color intensity represents magnitude of electric field). We also simulate the symmetric surface acoustic wave at the location of the interdigital transducer (IDT) which is shown by the aluminum (Al) electrodes in cross-section view (blue indicates highest magnitude of displacement, red indicates lowest displacement). (c) A photograph of the fabricated LiNbO_3_ device is presented. The IDT is angled to produce acoustic wave momentum along the clockwise (cw) circulation direction while an acoustic reflector helps to form a transverse standing wave. Some e-beam resist residue associated with fabrication is apparent on the surface. Port 1 and Port 2 indicate the grating couplers by which light is coupled into the waveguide.

Our specific resonator supports both TE_00_ and TE_10_ modes, as shown in Figure 2a, although other modes may be used, with the undesirable Rayleigh backscattering between cw and ccw circulations introduced as coupling terms *V*_1_ and *V*_2_. We ensure that these optical modes are close together in frequency space but have large separation in momentum space. Propagating phonons having frequency and momentum that bridge the gap between these optical modes can be introduced via piezoelectric excitation. The direction of the phonon propagation sets which circulation of optical modes is hybridized through acousto-optic scattering and breaks symmetry in the system. Here, the acousto-optic scattering process is enabled by moving boundary effect, photo-elastic effect and piezoelectrically induced electro-optic effect and details on these mechanisms can be found in Supplementary §S1 and citations [32], [33]. We note that there is also a necessary index texture required to ensure a non-zero overlap integral for the acousto-optic scattering process, the details of which can be found in the Supplementary §S2. With sufficiently large acousto-optic scattering rate (i.e., $G_{ph}>\sqrt{\kappa_{1}\kappa_{2}}$ ) the optical modes hybridize only for the phase-matched circulation direction around the device and produce a mode splitting phenomenon, which alters the spectral characteristics. The optical modes remain unchanged for the non-phase matched circulation direction, leading to a substantial reduction in spectral overlap between counter-propagating modes. This effectively suppresses the possibility of back-scattering events, even in the presence of scatterers.

We developed our telecom wavelength (∼1550 nm) experimental demonstration on a LiNbO_3_-on-insulator (LNOI) integrated photonics platform since the large piezoelectric coefficients of LiNbO_3_ enable the efficient excitation of surface acoustic waves and produce a significant acousto-optic coupling rate. The LNOI platform is also convenient for realizing integrated WGRs with relatively large optical Q-factors $\left(>10^{6}\right)$ . This combination of high optical Q-factors and large acousto-optic coupling rates promotes the realization of strong mode hybridization in our optical devices, thus enabling chiral dispersion as was demonstrated in [26]. As described above, we design and fabricate (Figure 2c) a racetrack resonator supporting TE_10_ and TE_00_ modes (Figure 2b and c) which can act as a two-level photonic molecule. We access the resonator with a single-mode waveguide in shunt configuration and add grating couplers to each end of this waveguide to provide off-chip optical access to the device. An aluminum interdigital transducer (IDT) is co-fabricated with orientation along the Y-30° direction of the lithium niobate crystal [34] to maximize the electromechanical coupling rate to the symmetric surface acoustic mode (Figure 2b). The phonons produced by the actuator propagate along a portion of the racetrack with momentum corresponding to the cw circulation direction. The actuator design includes an acoustic reflector to produce a transverse standing wave for producing the necessary acoustic wave texture [26]. The optical ports are explicitly labeled 1,2 in Figure 2c such that the *S*_21_ (*S*_12_) measurement through the waveguide reads the ccw (cw) optical states of the resonator. Importantly, the acousto-optic coupling is phase matched only in one direction, i.e., for the selected modes it is engaged only in optical *S*_21_ transmission measurements from port 1 to port 2 corresponding to the ccw circulation direction around the resonator.

**Figure 3:**
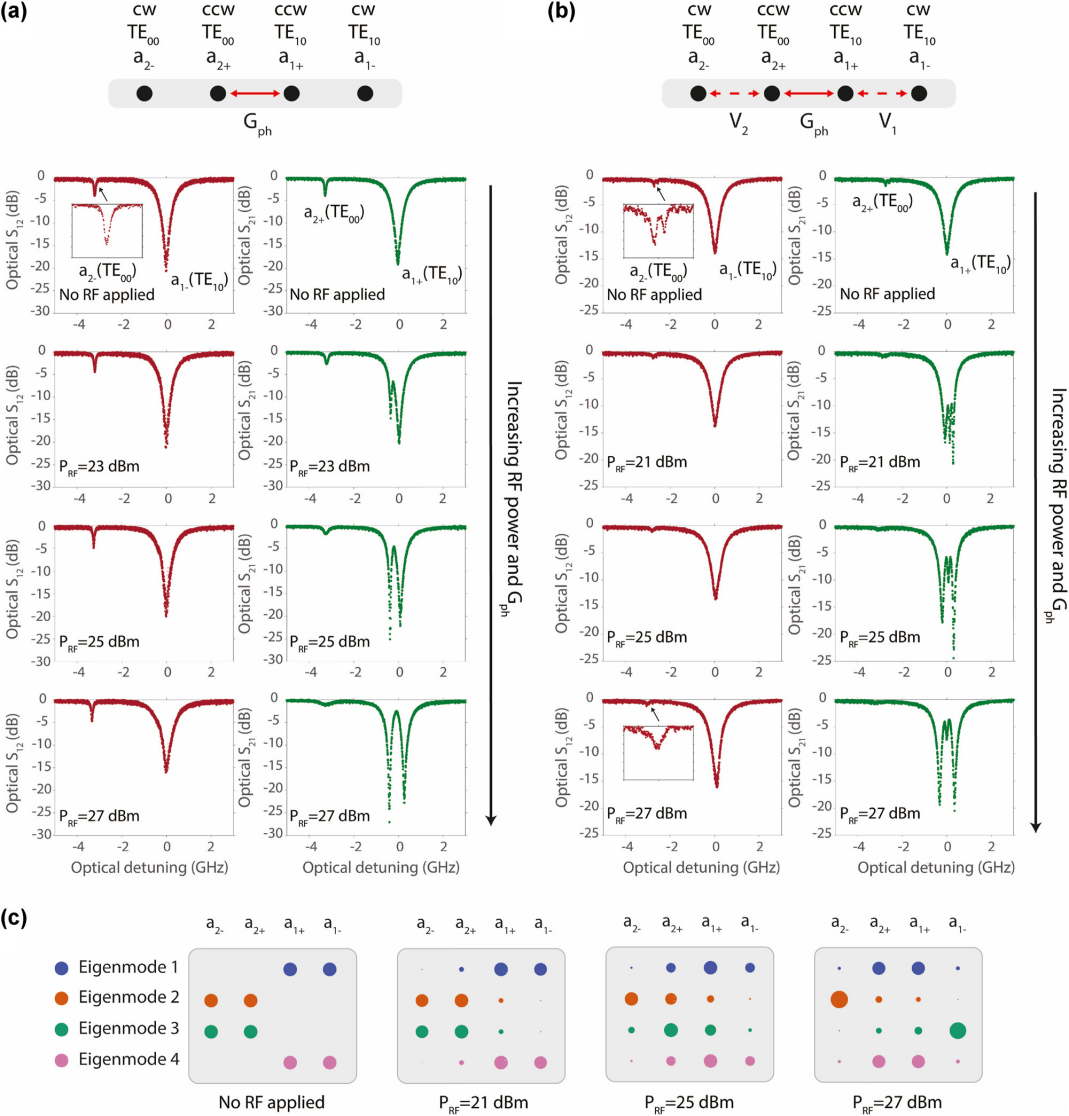
Experimental demonstration of phonon-induced chiral dispersion and the localization of cw modes. (a, b) Measured through-waveguide transmission (S-parameters *S*_21_, *S*_12_ are described in the text) for devices having (a) negligible backscattering and (b) significant backscattering. The insets show the zoomed-in versions of the TE_00_ modes in the cw circulation direction. Numerical quantification of the linewidths and scattering rates is presented in Supplementary Section §S8. In the top row we present a 1D chain equivalent of these two cases with mapping as described in the Supplementary §S4. The key observation of the suppression of backscattering is made in the insets of (b), where we observe the cw TE_00_ mode recover from a doublet into a single state when the TRS-breaking RF stimulus is provided to the IDT. (c) Simulated eigenvectors for the device in (b) under a rotating frame of reference described in §S4. With increasing acousto-optic coupling *G*_*ph*_ we observe a topological transition that leads to the emergence of protected boundary modes (Eigenmode 2,3) as explained in the manuscript.

In Figure 3, we present our experimental results for two devices that show both negligible (Figure 3a) and significant (Figure 3b) backscattering. The backscattering is unintentional and its source is not definitive, although it is speculated to originate either from device geometry defects or from the e-beam resist residue visible on the surface. We use a heterodyne detection system (Supplementary Section §S6) to probe the optical signal transmissions. With no RF power applied (top data row of Figure 3a and b), we observe that both optical transmission spectra show two distinct dips corresponding to the TE_00_ and TE_10_ modes of the resonator. The TE_10_ mode shows a more significant dip than the TE_00_ mode due to a larger evanescent field that increases coupling to the waveguide. The distinction in backscattering rate can be experimentally observed from the top insets in Figure 3a and b. The device with significant backscattering (Figure 3b) shows the characteristic doublet for the TE_00_ mode, which is in stark contrast to the case with negligible backscattering (Figure 3a). Due to the larger intrinsic loss rate, backscattering occurring within the TE_10_ mode is not manifested as a direct mode splitting but is instead revealed as some amount of linewidth broadening and an additional back-reflection from the resonator. We can now introduce photon–photon coupling in both systems by electrically launching phonons via the co-fabricated IDT. We specifically use a surface acoustic wave near 3 GHz, as simulated in Figure 2b, since its frequency and momentum best match the *ω* − *k* spacing of the TE_00_ and TE_10_ modes, and its shape provides the best overlap integral for acousto-optic scattering. A discussion of the transducer RF reflection spectrum is presented in the Supplementary §S7.

In Figure 3a, we show the evolution of the optical transmission through the device having negligible backscattering as a function of increasing RF input power (i.e., increasing acousto-optic coupling rate). In the phase-matched direction (the optical *S*_21_), the optical modes significantly hybridize, and as a result the TE_10_ mode shows a frequency splitting behavior proportional to the acousto-optic coupling rate. On the other hand, the optical modes remain unperturbed in the non-phase matched direction (the optical *S*_12_), thus demonstrating the chiral dispersion we wish to utilize.

We now look more closely at the device with higher backscattering, shown in Figure 3b. The evolution of the TE_10_ mode pair (i.e., cw and ccw) with increasing acousto-optic coupling shows that, even as the mode experiences a *G*_*ph*_-induced splitting in the phase (ccw) matched direction, a smaller third state can be seen peeking through at the original frequency. This extra state is apparent due to the disorder-induced backscattering (*V*_2_) into the ccw TE_10_ mode. A detailed analysis is presented in Supplementary §S2, Eqn. S(29). This extra state diminishes as the acousto-optic coupling *G*_*ph*_ surpasses the backscattering rates *V*_1_, *V*_2_, and should eventually disappear since the cw and ccw modes are no longer spectrally overlapping.

At this point it is informative to consider the topological configuration of the system of modes, by unwrapping *ω* − *k* diagram of Figure 2a into a flattened 1D chain. We can accomplish this using a frame of reference rotating with the acoustic pump frequency Ω (see Supplementary §S4), producing the 1D chains shown in Figure 3a and b. We can then compute the eigenvectors of the system, as shown in Figure 3c, to evaluate how the modes will evolve at various RF/acoustic pump powers. With no acousto-optic coupling the spatial profiles of all four eigenvectors show only the hybridization between forward and backward modes. With increased RF power, however, the forward and backward modes begin to diffuse to all other sites (see cases for 21 dBm and 25 dBm). At very large RF power (the 27 dBm case) we observe significant localization of the backward propagating modes. This phenomenon can be readily understood in terms of the topological Su–Schrieffer–Heeger (SSH) model for dimerized chains [35]. Here the backscattering *V*_1_, *V*_2_ takes the role of the intra-dimer bond while the acousto-optical modulation *G*_*ph*_ takes the role of the inter-dimer bond. In this analogy, familiar readers will see how exponentially localized edge states must emerge (labeled as Eigenmodes 2 and 3) when *G*_*ph*_ ≫ *V*_1_, *V*_2_ since the chain transforms into the topologically non-trivial phase. Importantly, we can discover from this topological viewpoint that the exact values of *G*_*ph*_ and *V*_1,2_ are not so important, and that the localization and mode recovery occurs as long as the non-trivial topological phase has been achieved. A higher *G*_*ph*_ leads to improved mode localization along the chain, and with it, recovery of the mode’s optical properties. For sufficiently large *G*_*ph*_ the modes on the chain edge will recover to their original linewidth and Rayleigh scattering will be completely suppressed. Additional discussion on the analogy to the SSH model and possible extension to a longer SSH chain can be found in the Supplementary §S5. Experimentally, we can confirm this effect when observing the ccw TE_00_ mode labeled *a*_2−_ in the *S*_12_ spectrum in Figure 3b – the inset shows clearly that the mode has recovered to a simple Lorentzian (i.e., the effective linewidth improves significantly) indicating also that the backscattering has been inhibited.

**Figure 4:**
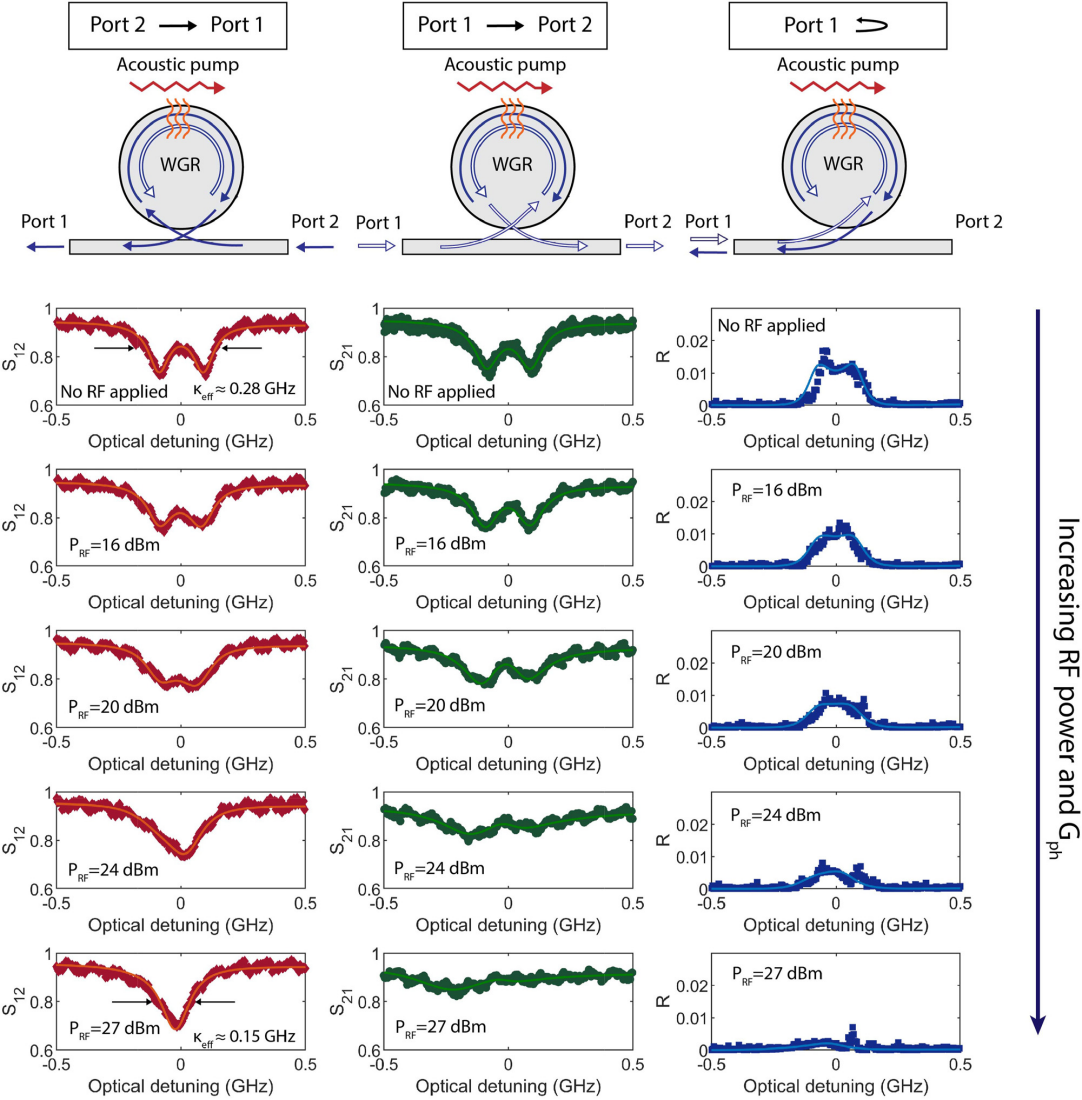
Experimental demonstration of the recovery of the TE_00_ mode and elimination of the disorder-induced reflection. The experimentally measured optical *S* parameters are presented (reflection coefficient *R* = *S*_11_ = *S*_22_) near the TE_00_ mode for a device having high rate of disorder-induced backscattering (all relevant device parameters can be found in Supplementary Section §S8). As before the *S*_21_ measurements (middle column) show the transmission associated with ccw phase-matched directionality within the resonator. We see that as the acousto-optic scattering rate *G*_*ph*_ increases, there is further a splitting of the mode induced in the ccw direction, leading to an internal chiral density of states. As a consequence, we simultaneously observe that in the cw direction (*S*_12_ measurements in first column) the optical mode recovers significantly from the undesirable doublet state back to a singlet Lorentzian shape – even though we did not perform any action to influence the mode in this direction – and additionally the reflection (third column) from the resonator is eliminated. Both pieces of evidence point to a suppression of Rayleigh backscattering within the resonator.

We now explicitly show the reduction in backscattered light and near-complete mode recovery within a device having a much larger rate of disorder-induced backscattering. To do so, we also modify our experimental setup by adding a circulator between the laser and the WGR, i.e. before Port 1, to enable simultaneous measurement of light back-reflected from within the resonator (Supplementary Section §S6). Experimental data are presented in Figure 4, showing forward transmission, reverse transmission, and back-reflection measurements through the waveguide. Only a single reflection measurement is necessary since the reflected signals for the forward and backward propagating directions are the same (see Supplementary §S3). We focus here on the TE_00_ mode as its linewidth is comparable with the backscattering rate, and it presents a clear visual of the undesirable mode splitting. The experiment was performed with a TE_00_/TE_10_ mode pair with initial frequency separation of 3.2 GHz. The RF input to the transducer is set at 3 GHz since that was experimentally determined to be the frequency that produces the largest acousto-optic coupling in this device. In spite of this frequency mismatch we do observe a significant phonon-induced hybridization of the forward optical states. This can be discerned from the further splitting of the doublet in the cw phase-matched direction. From fitting the experimental data (all numerical quantities involved with our experiments are in Supplementary Section §S8) we estimate that the intrinsic linewidth of the TE_00_ mode is ≈0.12 GHz, and the backscattering rates are *V*_1_ ≈ 0.17 GHz and *V*_2_ ≈ 0.27 GHz following the previous convention in Figures 2 and 3. The highest RF drive level tested was up to 27 dBm which we estimate corresponds to a *G*_*ph*_ ≈ 0.87 GHz, and is limited by the power handling capability of the IDT. At this point *G*_*ph*_ ≫ *V*_1_, *V*_2_ and the TE_00_ mode is seen to recover to a Lorentzian with ≈0.15 GHz linewidth, which is an almost complete recovery to the intrinsic loss rate of 0.12 GHz. Most importantly, we note a significant reduction in the back-reflected light returned to port 1, which is a direct observation confirming near-complete suppression of disorder-induced Rayleigh backscattering is suppressed within the WGR.

## 3 Conclusions

Disorder-induced backscattering is a considerable technical challenge for integrated photonics platforms. Our experiments demonstrate that introducing chiral dispersions via time-reversal symmetry breaking integrated wave-guiding structures – here, a resonator – can suppress this unwanted scattering effect even when disorder and defects may be present. We confirm this experimentally by studying the optical states of our resonators and by directly observing the reduction in backscattered light. We additionally provide a new topological explanation for the phenomenon. This is an extremely important capability since isolators and circulators can only discard back-reflected photons and does nothing to suppress the scattering from taking place at its point of origination. While our specific technique does require a sacrifice of the modes in a counter-propagating direction, there is still tremendous potential for systems where unidirectional light transport is necessary. For instance, the application of such a technique on a non-resonant waveguide could ensure very high fidelity unidirectional light transport from an integrated laser to the rest of the optical system, without requiring an isolator. Backscattering free inter-chip light transport might also be accomplished by similarly breaking TRS across a butt coupled waveguide-waveguide interface. Finally, we note that our acousto-optic approach in lithium niobate is by no means exclusive, and there are plenty of alternatives that could offer good solutions to suppressing disorder-induced backscattering in integrated photonics. These include light-sound interactions [19], [20], [21], [22], [23], [24], [25], [26], electro-optic interaction [27], [28], [29], and on-chip magneto-optic devices [36], [37], [38], [39].

## Supplementary Material

Supplementary Material Details
