# Supplementary material for: Electrically-controlled suppression of Rayleigh backscattering in an integrated photonic circuit
Source: Nanophotonics. 2024 Jan 8;13(2):173–81. doi: 10.1515/nanoph-2023-0431 (PMC11501930; doi:10.1515/nanoph-2023-0431)
Supplement: Supplementary file 1 — Supplementary Material Details [file j_nanoph-2023-0431_suppl_001.pdf]

Supplementary Information: Electrically-Controlled Suppression of Rayleigh Backscattering in an Integrated Photonic Circuit

Oğulcan E. Örsel [1], Jiho Noh [2], and Gaurav Bahl [2]

[1] Department of Electrical & Computer Engineering,
[2] Department of Mechanical Science and Engineering,
University of Illinois at Urbana–Champaign, Urbana, IL 61801 USA,

S1 Estimation of acousto-optic interaction strength

The acousto-optic coupling rate between two optical modes can calculated by taking into account various factors including the influence of a moving boundary, as well as the effects of photoelasticity and electro-optics. In the context of the photoelastic effect, the relation between the change in the optical index and the induced strain is characterized by a fourth-order tensor. Its mathematical representation is given by [S1, S2]:

$$\begin{bmatrix} \Delta B_1 \\ \Delta B_2 \\ \Delta B_3 \\ \Delta B_4 \\ \Delta B_5 \\ \Delta B_6 \end{bmatrix} = \begin{bmatrix} p_{11} & p_{12} & p_{13} & 0 & 0 & 0 \\ p_{12} & p_{11} & p_{13} & 0 & 0 & 0 \\ p_{13} & p_{13} & p_{33} & 0 & 0 & 0 \\ 0 & 0 & 0 & p_{44} & 0 & 0 \\ 0 & 0 & 0 & 0 & p_{44} & 0 \\ 0 & 0 & 0 & 0 & 0 & p_{66} \end{bmatrix} \begin{bmatrix} S_1 \\ S_2 \\ S_3 \\ S_4 \\ S_5 \\ S_6 \end{bmatrix} \tag{S1}$$

In this equation, ΔB_i signifies the change in the optical indicatrix, p_{ij} stands for the photoelastic tensor, and S_j represents the in-plane strain resulting from the acoustic wave. To calculate the perturbation in the effective refractive index, we utilize the first-order perturbation theory [S1, S2]:

$$\Delta n_{\mathrm{EO}} = -\frac{\epsilon_0 n^5}{2} \frac{\int \left[\boldsymbol{\mathcal{E}}_2(\boldsymbol{r}_\perp)\right]^H \begin{bmatrix} \Delta B_1 & \Delta B_6 & \Delta B_5 \\ \Delta B_6 & \Delta B_2 & \Delta B_4 \\ \Delta B_5 & \Delta B_4 & \Delta B_3 \end{bmatrix} \left[\boldsymbol{\mathcal{E}}_1(\boldsymbol{r}_\perp)\right] d\boldsymbol{r}_\perp + c.c.}{\int (\boldsymbol{\mathcal{E}}_2(\boldsymbol{r}_\perp) + \boldsymbol{\mathcal{E}}_1(\boldsymbol{r}_\perp)) \cdot (\boldsymbol{\mathcal{D}}_2(\boldsymbol{r}_\perp) + \boldsymbol{\mathcal{D}}_1(\boldsymbol{r}_\perp))\, d\boldsymbol{r}_\perp} \tag{S2}$$

Here, ϵ_0 stands for vacuum permittivity, n denotes the refractive index, $\boldsymbol{\mathcal{E}}_1(\boldsymbol{r}\perp)$ and $\boldsymbol{\mathcal{E}}_2(\boldsymbol{r}\perp)$ ($\boldsymbol{\mathcal{D}}_1(\boldsymbol{r}\perp)$ and $\boldsymbol{\mathcal{D}}_2(\boldsymbol{r}\perp)$) represent the transverse electric (displacement) field profiles of the optical modes, and H corresponds to the Hermitian

operator. Furthermore, we can write

$$\left[\boldsymbol{\mathcal{E}}_2(\boldsymbol{r}_\perp)\right] = \begin{bmatrix} \mathcal{E}_2^x(\boldsymbol{r}_\perp) \\ \mathcal{E}_2^y(\boldsymbol{r}_\perp) \\ \mathcal{E}_2^z(\boldsymbol{r}_\perp) \end{bmatrix} \quad \text{and} \quad \left[\boldsymbol{\mathcal{E}}_1(\boldsymbol{r}_\perp)\right] = \begin{bmatrix} \mathcal{E}_1^x(\boldsymbol{r}_\perp) \\ \mathcal{E}_1^y(\boldsymbol{r}_\perp) \\ \mathcal{E}_1^z(\boldsymbol{r}_\perp) \end{bmatrix} \tag{S3}$$

Similarly, the moving boundary effect phenomenon emerges from the change in the effective optical index resulting from variations in waveguide dimensions due to mechanical strain. This effect is calculated by the following overlap integral [S1, S2]:

$$\Delta n_{MB} = -\frac{\epsilon_0 n}{2} \frac{\oint (Q \cdot \widehat{n})(\boldsymbol{\mathcal{E}}_{2,\parallel}^H(\boldsymbol{r}_\perp)\Delta\epsilon \boldsymbol{\mathcal{E}}_{1,\parallel}(\boldsymbol{r}_\perp) - \boldsymbol{\mathcal{D}}_{2,\perp}^H(\boldsymbol{r}_\perp)\Delta\epsilon^{-1}\boldsymbol{\mathcal{D}}_{1,\perp}(\boldsymbol{r}_\perp) d\boldsymbol{S} + c.c.}{\int (\boldsymbol{\mathcal{E}}_2(\boldsymbol{r}_\perp) + \boldsymbol{\mathcal{E}}_1(\boldsymbol{r}_\perp)) \cdot (\boldsymbol{\mathcal{D}}_2(\boldsymbol{r}_\perp) + \boldsymbol{\mathcal{D}}_1(\boldsymbol{r}_\perp))\, d\boldsymbol{r}_\perp} \tag{S4}$$

Here, Q signifies the mechanical displacement field, while $\widehat{n}$ indicates the surface normal pointing from the high-index to the low-index media. $\boldsymbol{\mathcal{E}}_{i,\parallel}(\boldsymbol{r}\perp)$ stands for the electric field parallel to the waveguide's surface, and $\boldsymbol{\mathcal{D}}_{i,\perp}(\boldsymbol{r}\perp)$ corresponds to the electric displacement field perpendicular to the waveguide's surface. Additionally, we define $\Delta\epsilon = \epsilon_{\text{core}} - \epsilon_{\text{clad}}$ and $\Delta\epsilon^{-1} = \epsilon_{\text{core}}^{-1} - \epsilon_{\text{clad}}^{-1}$, where ϵ_{core} and ϵ_{clad} represent the permittivity of the core and cladding materials, respectively. Moreover, the acoustic wave gives rise to an associated electric field due to the piezoelectric effect. This electric field also induces a change in the indicatrix, which is expressed as:

$$\begin{bmatrix} \Delta B_1 \\ \Delta B_2 \\ \Delta B_3 \\ \Delta B_4 \\ \Delta B_5 \\ \Delta B_6 \end{bmatrix} = \begin{bmatrix} 0 & -r_{22} & r_{31} \\ 0 & r_{22} & r_{31} \\ 0 & 0 & r_{33} \\ 0 & r_{42} & 0 \\ r_{42} & 0 & 0 \\ -r_{22} & 0 & 0 \end{bmatrix} \begin{bmatrix} \mathcal{E}_b^x(\boldsymbol{r}_\perp) \\ \mathcal{E}_b^y(\boldsymbol{r}_\perp) \\ \mathcal{E}_b^z(\boldsymbol{r}_\perp) \end{bmatrix} \tag{S5}$$

$r_{i,j}$ stands for the electro-optic coefficients, and $\mathcal{E}_b^j(\boldsymbol{r}\perp)$ represents the transverse profile of the electric field induced by the piezoelectric effect. Likewise, we can determine the change in the refractive index by applying the perturbation theorem, resulting in:

$$\Delta n_{\text{EO}} = -\frac{\epsilon_0 n^5}{2} \frac{\int \left[\boldsymbol{\mathcal{E}}_2(\boldsymbol{r}_\perp)\right]^H \begin{bmatrix} \Delta B_1 & \Delta B_6 & \Delta B_5 \\ \Delta B_6 & \Delta B_2 & \Delta B_4 \\ \Delta B_5 & \Delta B_4 & \Delta B_3 \end{bmatrix} \left[\boldsymbol{\mathcal{E}}_1(\boldsymbol{r}_\perp)\right] d\boldsymbol{r}_\perp + c.c.}{\int (\boldsymbol{\mathcal{E}}_2(\boldsymbol{r}_\perp) + \boldsymbol{\mathcal{E}}_1(\boldsymbol{r}_\perp)) \cdot (\boldsymbol{\mathcal{D}}_2(\boldsymbol{r}_\perp) + \boldsymbol{\mathcal{D}}_1(\boldsymbol{r}_\perp))\, d\boldsymbol{r}_\perp} \tag{S6}$$

By combining various perturbation sources like the photoelastic effect, moving boundary effect, and electro-optic effect, we can then compute the collective change in the refractive index resulting from the acousto-optic interaction as:

$$\Delta n = \Delta n_{PE} + \Delta n_{MB} + \Delta n_{EO} \tag{S7}$$

Finally, we can calculate the acousto-optic coupling rate for a resonator as [S2]:

$$G_{ph} = \frac{\omega_0 \Delta n \eta}{n_{\mathrm{g}}} \tag{S8}$$

Here, ω_0 is the optical frequency, $n_g = \sqrt{n_g^1 n_g^2}$ represents the geometric mean of the group indices of the modes (More detailed explanation can be found in [S2]), and η is the fraction of the resonator that is under the acousto-optic modulation. During the device design phase, we aimed to achieve the highest acousto-optic coupling rate by considering both the electro-mechanical conversion efficiency of the IDTs and the overlap integrals within the equations S2, S4 and S6. Consequently, we opted for X-cut $LiNbO_3$ as our material choice and positioned our IDTs along the Y-30° direction for optimal results. In Fig.S1, we illustrate a specific instance of acousto-optic coupling rate computation in this particular configuration, showcasing distinct contributing factors. Here, we see that the strongest contribution comes from the piezoelectrically induced electro-optic effect, and the overall acousto-optic coupling rate maximizes around 3 GHz where the efficient acoustic excitation occurs.

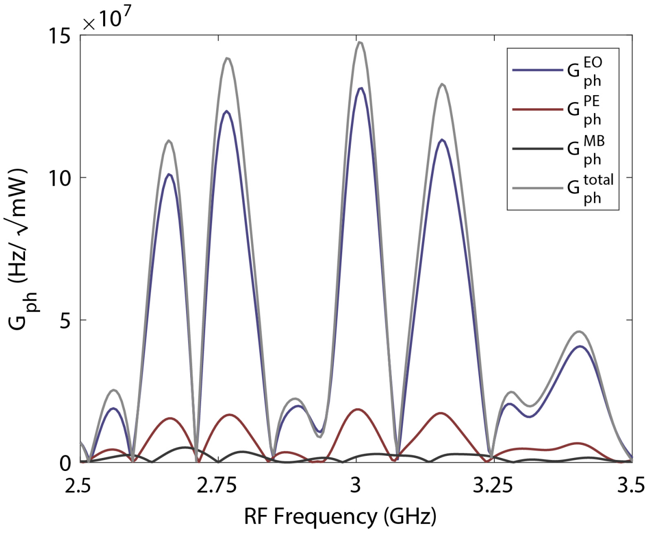

Figure S1: Simulated acousto-optic coupling rate. The total acousto-optic coupling rate incorporates various sources, including the moving boundary effect, photo-elastic effect, and the electro-optic effect. Among these, the electro-optic effect emerges as the dominant contributor, reaching its peak at approximately 3 GHz, aligning with the point of optimal acoustic excitation.

S2 Two-level photonic system with acousto-optic and Rayleigh interaction

Our photonic system consists of two optical modes, TE_{00} (ω_2, k_2) and TE_{10} (ω_1, k_1) that are supported within a racetrack resonator. These modes are coupled with an acoustic pump (Ω, q) that bridges the frequency ($\Omega = \omega_1 - \omega_2$) and momentum ($q = k_2 - k_1$) gap between them. Since this coupling (or the three-wave mixing process) is satisfied in a unidirectional manner, we use the term "Forward" for the phase-matched direction and "Backward" for the non-phase-matched direction. For the forward direction with $\Omega = \omega_1 - \omega_2$, we can then write the interaction Hamiltonian for the acousto-optic interaction as [S3–S6];

$$H_{in}^{AO} = \hbar \left(\frac{g_{ph}}{2} \hat{a}_{1+}^{\dagger} \hat{a}_{2+} \hat{b} + \frac{g_{ph}^{*}}{2} \hat{a}_{1+} \hat{a}_{2+}^{\dagger} \hat{b}^{\dagger} \right) \tag{S9}$$

We define the single photon acousto-optic coupling rate between the modes as $g_{\text{ph}} \propto \delta(k_2 - k_1 - q) \int \mathcal{E}_1(\mathbf{r}_\perp)\mathcal{E}_2(\mathbf{r}_\perp) u(\mathbf{r}_\perp) d\boldsymbol{r}_\perp$ [S3], where $\mathcal{E}_1(\boldsymbol{r}_\perp)$, $\mathcal{E}_2(\boldsymbol{r}_\perp)$ and $u(\boldsymbol{r}_\perp)$ are the transverse mode profiles of the optical and acoustic modes, respectively. Here $\delta(k_2 - k_1 - q)$ represents the unidirectional phase matching feature of the process, $\hat{a}_{1+}^{\dagger}(\hat{a}_{1+})$, $\hat{a}_{2+}^{\dagger}(\hat{a}_{2+})$ and $\hat{b}^{\dagger}(\hat{b})$ represent creation (annihilation) operators for TE_{10}, TE_{00} photons and acoustic phonons respectively. In addition to the acousto-optic coupling rate, we consider the interaction of the forward optical modes, TE_{00+} ($\hat{a}_{2+}$) and TE_{10+} ($\hat{a}_{1+}$), with their time-reversal counterparts, $\text{TE}_{00\text{-}}$ ($\hat{a}_{2-}$) and $\text{TE}_{10\text{-}}$ ($\hat{a}_{1-}$), as well. The coupling is induced due to surface roughness or internal inhomogeneities and is usually referred to as Rayleigh scattering. Under the dipole approximation, we can write the interaction Hamiltonian for this case as,

$$H_{in}^{R} = \hbar \frac{V_1}{2} \left(\hat{a}_{1+}^{\dagger} \hat{a}_{1-} + \hat{a}_{1-}^{\dagger} \hat{a}_{1+} \right) + \hbar \frac{V_2}{2} \left(\hat{a}_{2+}^{\dagger} \hat{a}_{2-} + \hat{a}_{2-}^{\dagger} \hat{a}_{2+} \right) \tag{S10}$$

Here we define V_1 and V_2 as the backscattering rates for the TE_{10} and TE_{00} modes, respectively. These coupling rates depend on the effective mode volume, the overlap of the scatterer with the optical mode, and the optical frequency. For our case, the optical modes are expected to show different backscattering rates, and this is verified by the experiments.

Having defined the interaction Hamiltonian for both cases, we can now write the Heisenberg-Langevin equations of our system. While doing so, we also treat the Heisenberg-Langevin equations classically by making substitutions as follows: $\hat{a}_{1+}(\hat{a}_{1-}) \to a_{1+}(a_{1-})$, $\hat{a}_{2+}(\hat{a}_{2-}) \to a_{2+}(a_{2-})$ and $\hat{b}\, g_{ph}/2 \to b\, g_{ph}/2$. Here, $a_{1+}(a_{1-})$ and $a_{2+}(a_{2-})$ are intracavity field amplitudes of the forward (backward) TE_{10} and TE_{00} modes respectively. Also, b represents the steady-state amplitude of the acoustic wave under non-depleted RF pump approximation. Furthermore, we also combine b with the single photon acousto-optic coupling rate and re-write it as $G_{ph}/2$. Then, the equations of motion become:

$$\partial_t \begin{pmatrix} a_1^+ \\ a_2^+ \\ a_1^- \\ a_2^- \end{pmatrix} = \begin{pmatrix} -\frac{\kappa_1}{2} - i\omega_1 & -i\frac{G_{ph}}{2}e^{-i\Omega t} - \Gamma_c & -i\frac{V_1}{2} & 0 \\ -i\frac{G_{ph}}{2}e^{i\Omega t} - \Gamma_c & -\frac{\kappa_2}{2} - i\omega_2 & 0 & -i\frac{V_2}{2} \\ -i\frac{V_1}{2} & 0 & -\frac{\kappa_1}{2} - i\omega_1 & -i\frac{G_{ph}}{2}e^{i\Omega t} - \Gamma_c \\ 0 & -i\frac{V_2}{2} & -i\frac{G_{ph}}{2}e^{-i\Omega t} - \Gamma_c & -\frac{\kappa_2}{2} - i\omega_2 \end{pmatrix} \begin{pmatrix} a_1^+ \\ a_2^+ \\ a_1^- \\ a_2^- \end{pmatrix} + \begin{pmatrix} \sqrt{\kappa_{ex1}} s_{in}^+ \\ \sqrt{\kappa_{ex2}} s_{in}^+ \\ \sqrt{\kappa_{ex1}} s_{in}^- \\ \sqrt{\kappa_{ex2}} s_{in}^- \end{pmatrix} \tag{S11}$$

Here κ_1 (κ_2) and κ_{ex1} (κ_{ex2}) represent the total loss rate and the external coupling rate of the TE_{10} (TE_{00}) modes. ω_1 (ω_2) is the resonant frequency of TE_{10} (TE_{00}) mode, and G_{ph} is the phonon enhanced acousto-optic coupling rate. We also added $\Gamma_c = \sqrt{\kappa_{ex1}\kappa_{ex2}}/2$ [S7], which describes the coupling between TE_{00} and TE_{10} modes due to the presence of the probe waveguide.

To solve the above equation S11, we need to take the Fourier transform of both sides. Since our system is a linear time-varying system, we first need to decompose the optical fields into Fourier components. For that purpose, we consider the TE_{10} mode to be located at a higher frequency than the TE_{00} mode and expand the Fourier components as,

$$a_1^+ = a_{1,0}^+ e^{-i\omega_l t} + a_{1,+1}^+ e^{-i(\omega_l + \Omega)t} \tag{S12}$$

$$a_2^+ = a_{2,0}^+ e^{-i\omega_l t} + a_{2,-1}^+ e^{-i(\omega_l - \Omega)t} \tag{S13}$$

$$a_1^- = a_{1,0}^- e^{-i\omega_l t} + a_{1,-1}^- e^{-i(\omega_l - \Omega)t} \tag{S14}$$

$$a_2^- = a_{2,0}^- e^{-i\omega_l t} + a_{2,+1}^- e^{-i(\omega_l + \Omega)t} \tag{S15}$$

We then substitute these equations into equation S11 to obtain three sets of matrix equations at different frequencies. At the optical carrier frequency ω_l, we have :

$$\partial_t \begin{pmatrix} a_{1,0}^+ e^{-i\omega_l t} \\ a_{2,0}^+ e^{-i\omega_l t} \\ a_{1,0}^- e^{-i\omega_l t} \\ a_{2,0}^- e^{-i\omega_l t} \end{pmatrix} = \begin{pmatrix} -\frac{\kappa_1}{2} - i\omega_1 & -\Gamma_c & -i\frac{V_1}{2} & 0 \\ -\Gamma_c & -\frac{\kappa_2}{2} - i\omega_2 & 0 & -i\frac{V_2}{2} \\ -i\frac{V_1}{2} & 0 & -\frac{\kappa_1}{2} - i\omega_1 & -\Gamma_c \\ 0 & -i\frac{V_2}{2} & -\Gamma_c & -\frac{\kappa_2}{2} - i\omega_2 \end{pmatrix} \begin{pmatrix} a_{1,0}^+ e^{-i\omega_l t} \\ a_{2,0}^+ e^{-i\omega_l t} \\ a_{1,0}^- e^{-i\omega_l t} \\ a_{2,0}^- e^{-i\omega_l t} \end{pmatrix} + \begin{pmatrix} 0 & -i\frac{G_{ph}}{2} & 0 & 0 \\ -i\frac{G_{ph}}{2} & 0 & 0 & 0 \\ 0 & 0 & 0 & -i\frac{G_{ph}}{2} \\ 0 & 0 & -i\frac{G_{ph}}{2} & 0 \end{pmatrix} \begin{pmatrix} a_{1,+1}^+ e^{-i\omega_l t} \\ a_{2,-1}^+ e^{-i\omega_l t} \\ a_{1,-1}^- e^{-i\omega_l t} \\ a_{2,+1}^- e^{-i\omega_l t} \end{pmatrix} + \begin{pmatrix} \sqrt{\kappa_{ex1}} s_{in}^+ e^{-i\omega_l t} \\ \sqrt{\kappa_{ex2}} s_{in}^+ e^{-i\omega_l t} \\ \sqrt{\kappa_{ex1}} s_{in}^- e^{-i\omega_l t} \\ \sqrt{\kappa_{ex2}} s_{in}^- e^{-i\omega_l t} \end{pmatrix} \tag{S16}$$

Similarly, at $\omega_l - \Omega$:

$$\partial_t \begin{pmatrix} 0 \\ a^+_{2,-1} e^{-i(\omega_l-\Omega)t} \\ a^-_{1,-1} e^{-i(\omega_l-\Omega)t} \\ 0 \end{pmatrix} = \begin{pmatrix} -\frac{\kappa_1}{2} - i\omega_1 & -\Gamma_c & -i\frac{V_1}{2} & 0 \\ -\Gamma_c & -\frac{\kappa_2}{2} - i\omega_2 & 0 & -i\frac{V_2}{2} \\ -i\frac{V_1}{2} & 0 & -\frac{\kappa_1}{2} - i\omega_1 & -\Gamma_c \\ 0 & -i\frac{V_2}{2} & -\Gamma_c & -\frac{\kappa_2}{2} - i\omega_2 \end{pmatrix}$$
$$\times \begin{pmatrix} 0 \\ a^+_{2,-1} e^{-i(\omega_l-\Omega)t} \\ a^-_{1,-1} e^{-i(\omega_l-\Omega)t} \\ 0 \end{pmatrix} + \begin{pmatrix} 0 & -i\frac{G_{ph}}{2} & 0 & 0 \\ -i\frac{G_{ph}}{2} & 0 & 0 & 0 \\ 0 & 0 & 0 & -i\frac{G_{ph}}{2} \\ 0 & 0 & -i\frac{G_{ph}}{2} & 0 \end{pmatrix} \begin{pmatrix} a^+_{1,0} e^{-i(\omega_l-\Omega)t} \\ 0 \\ 0 \\ a^-_{2,0} e^{-i(\omega_l-\Omega)t} \end{pmatrix} \tag{S17}$$

Similarly, we have at $\omega_l + \Omega$:

$$\partial_t \begin{pmatrix} a^+_{1,+1} e^{-i(\omega_l+\Omega)t} \\ 0 \\ 0 \\ a^-_{2,+1} e^{-i(\omega_l+\Omega)t} \end{pmatrix} = \begin{pmatrix} -\frac{\kappa_1}{2} - i\omega_1 & -\Gamma_c & -i\frac{V_1}{2} & 0 \\ -\Gamma_c & -\frac{\kappa_2}{2} - i\omega_2 & 0 & -i\frac{V_2}{2} \\ -i\frac{V_1}{2} & 0 & -\frac{\kappa_1}{2} - i\omega_1 & -\Gamma_c \\ 0 & -i\frac{V_2}{2} & -\Gamma_c & -\frac{\kappa_2}{2} - i\omega_2 \end{pmatrix}$$
$$\times \begin{pmatrix} a^+_{1,+1} e^{-i(\omega_l+\Omega)t} \\ 0 \\ 0 \\ a^-_{2,+1} e^{-i(\omega_l+\Omega)t} \end{pmatrix} + \begin{pmatrix} 0 & -i\frac{G_{ph}}{2} & 0 & 0 \\ -i\frac{G_{ph}}{2} & 0 & 0 & 0 \\ 0 & 0 & 0 & -i\frac{G_{ph}}{2} \\ 0 & 0 & -i\frac{G_{ph}}{2} & 0 \end{pmatrix} \begin{pmatrix} 0 \\ a^+_{2,0} e^{-i(\omega_l+\Omega)t} \\ a^-_{1,0} e^{-i(\omega_l+\Omega)t} \\ 0 \end{pmatrix} \tag{S18}$$

These 12 equations describe the state of our two-level photonic system under both acousto-optic and Rayleigh scattering. From these equations, we can derive the intracavity field vector at the carrier frequency (ω_l) as:

$$S = \begin{pmatrix} -\frac{\kappa_1}{2} - i\omega_1 & -\Gamma_c & -i\frac{V_1}{2} & 0 \\ -\Gamma_c & -\frac{\kappa_2}{2} - i\omega_2 & 0 & -i\frac{V_2}{2} \\ -i\frac{V_1}{2} & 0 & -\frac{\kappa_1}{2} - i\omega_1 & -\Gamma_c \\ 0 & -i\frac{V_2}{2} & -\Gamma_c & -\frac{\kappa_2}{2} - i\omega_2 \end{pmatrix} \tag{S19}$$

$$C_n = \begin{pmatrix} 0 & 0 & 0 & 0 \\ -i\frac{G_{ph}}{2} & 0 & 0 & 0 \\ 0 & 0 & 0 & -i\frac{G_{ph}}{2} \\ 0 & 0 & 0 & 0 \end{pmatrix}, C_p = \begin{pmatrix} 0 & -i\frac{G_{ph}}{2} & 0 & 0 \\ 0 & 0 & 0 & 0 \\ 0 & 0 & 0 & 0 \\ 0 & 0 & -i\frac{G_{ph}}{2} & 0 \end{pmatrix} \tag{S20}$$

$$\gamma_n = \left[-(\omega_l - \Omega)\mathbb{1} - S\right]^{-1} C_n \quad , \quad \gamma_p = \left[-(\omega_l + \Omega)\mathbb{1} - S\right]^{-1} C_p \tag{S21}$$

$$A_0 = \left[-\omega_l \mathbb{1} - S - C_p\gamma_n - C_n\gamma_p\right]^{-1} \begin{pmatrix} \sqrt{\kappa_{ex1}} s^+_{in} \\ \sqrt{\kappa_{ex2}} s^+_{in} \\ \sqrt{\kappa_{ex1}} s^-_{in} \\ \sqrt{\kappa_{ex2}} s^-_{in} \end{pmatrix} \tag{S22}$$

We then calculate the output spectrum at the carrier frequency as:

$$s_{out}^{+} = s_{in}^{+} - \begin{pmatrix} \sqrt{\kappa_{ex1}} \\ \sqrt{\kappa_{ex2}} \\ 0 \\ 0 \end{pmatrix}^{T} A_0 \tag{S23}$$

$$r_{out}^{+} = - \begin{pmatrix} 0 \\ 0 \\ \sqrt{\kappa_{ex1}} \\ \sqrt{\kappa_{ex2}} \end{pmatrix}^{T} A_0 \tag{S24}$$

$$s_{out}^{-} = s_{in}^{-} - \begin{pmatrix} 0 \\ 0 \\ \sqrt{\kappa_{ex1}} \\ \sqrt{\kappa_{ex2}} \end{pmatrix}^{T} A_0 \tag{S25}$$

$$r_{out}^{-} = - \begin{pmatrix} \sqrt{\kappa_{ex1}} \\ \sqrt{\kappa_{ex2}} \\ 0 \\ 0 \end{pmatrix}^{T} A_0 \tag{S26}$$

Here, we define $s_{in}^{+}(s_{in}^{-})$, $s_{out}^{+}(s_{out}^{-})$ and $r_{out}^{+}(r_{out}^{-})$ for a two port system (Port 1 and Port 2.). For an excitation at port 1 (port 2) with $s_{in}^{+}(s_{in}^{-})$, $s_{out}^{+}(s_{out}^{-})$ is the transmitted light at port 2 (port 1), and $r_{out}^{+}(r_{out}^{-})$ is the reflected light at port 1 (port 2).

For a better understanding of our model, we consider the experimental results given in Fig. 3. To simplify the analysis, we assume that the external coupling rate of the TE_{00} mode is approximately zero ($\kappa_{ext2} \approx 0$), and the modal loss rates (κ_1 and κ_2) are similar to each other with V_1 being ignored.

Also, we are interested in the optical output at the input (carrier) frequency $s_{out,0}$ (ignoring the sidebands $s_{out,1}$) for the phase-matched direction. We can then write:

$$s_{out,0}^{+} = s_{in}^{+} - \sqrt{\kappa_{ex1}}\, a_{1,0,+} \tag{S27}$$

where the TE_{10} intracavity field at the input frequency ($a_{1,0+}$), and it can be expressed as:

$$a_{1,0+} = \frac{\sqrt{\kappa_{ex1}} \left[\frac{V_2^2}{4} + \left(\frac{\kappa_1}{2} - i\Delta \right)^2 \right]}{\left[\frac{G_{ph}^2}{4} + \frac{V_2^2}{4} + \left(\frac{\kappa_1}{2} - i\Delta \right)^2 \right] \left(\frac{\kappa_1}{2} - i\Delta \right)} s_{in} \tag{S28}$$

Here, $\Delta = \omega_l - \omega_1$ is the optical detuning from the TE_{10} mode. We can then simplify the TE_{10} intracavity field to three diagonalized intracavity fields by assuming $G_{ph} \gg V_2$;

$$a_{1,0+} = \frac{V_2^2}{G_{ph}^2}\left(\frac{\sqrt{\kappa_{ex2}}}{\kappa_1/2 - i\Delta}\right) + \frac{G_{ph}^2 - V_2^2}{G_{ph}^2}\left[\frac{\sqrt{\kappa_{ex2}}/2}{\kappa_1/2 - i\left(\Delta + G_{ph}/2\right)}\right] + \frac{G_{ph}^2 - V_2^2}{G_{ph}^2}\left[\frac{\sqrt{\kappa_{ex2}}/2}{\kappa_1/2 - i\left(\Delta - G_{ph}/2\right)}\right] s_{in} \tag{S29}$$

Here we can easily see that the cavity susceptibility is now modified from a single Lorentzian response and instead exhibits three distinctive Lorentzian responses. If the acousto-optic coupling rate is large enough ($G_{ph} \gg \sqrt{\kappa_1\kappa_2}$ and $G_{ph} \gg V_2$), these responses correspond to the dressed states of TE_{10} mode and the backscattered TE_{00} mode, which coincides with the original TE_{10} in the forward direction. As apparent from equation S29, the cavity susceptibilities for the dressed states increase and split further in frequency when the G_{ph} increases. On the other hand, the cavity susceptibility of the backward TE_{00} mode decreases, demonstrating the suppression of the backscattering. In other words, the optical states for the forward modes change. This modification produces a reduction of the spectral overlap between the counter-propagating modes.

As discussed in the main text (Fig. 3b), the optical transmission measurement verifies this behavior as a reduced height for the central dip and increased splitting for the outer modes (i.e. the dressed states).

S3 Example evolution of transmission and reflection coefficients

Here, we consider an example case to understand the system dynamics under increasing acousto-optic coupling rate. For optical modes, we assume they have matched optical parameters: $\kappa_1 = \kappa_2 = 0.1$ GHz, $V_1/2 = V_2/2 = 0.15$ GHz, $\kappa_{ex1} = \kappa_{ex2} = 0.05$ GHz, $\omega_1 - \omega_2 = 5$ GHz and $\Omega = 5$ GHz. We plot only one of the modes since they have identical optical parameters. In Fig. S2, we present the optical S parameters of the system for increasing acousto-optic coupling rate (G_{ph}). For $G_{\mathrm{ph}} = 0$, the modes exhibit their intrinsic backscattering induced mode splitting, which is clearly resolved since the backscattering rate is larger than the optical loss rates. As we increase G_{ph}, we see that the mode in the non-phase matched direction recovers from this undesirable doublet state to a singlet state, just as is observed in our experiments. The mode in the phase-matched direction, i.e. where the G_{ph} is active, splits even further due to strong acousto-optic mode hybridization. The unwanted reflections due to intrinsic Rayleigh scattering also monotonically decrease. One crucial observation is that

the optical reflection coefficients are the same (optical S_{11}=S_{22}) for opposing directions due to the symmetry of the reflection process.

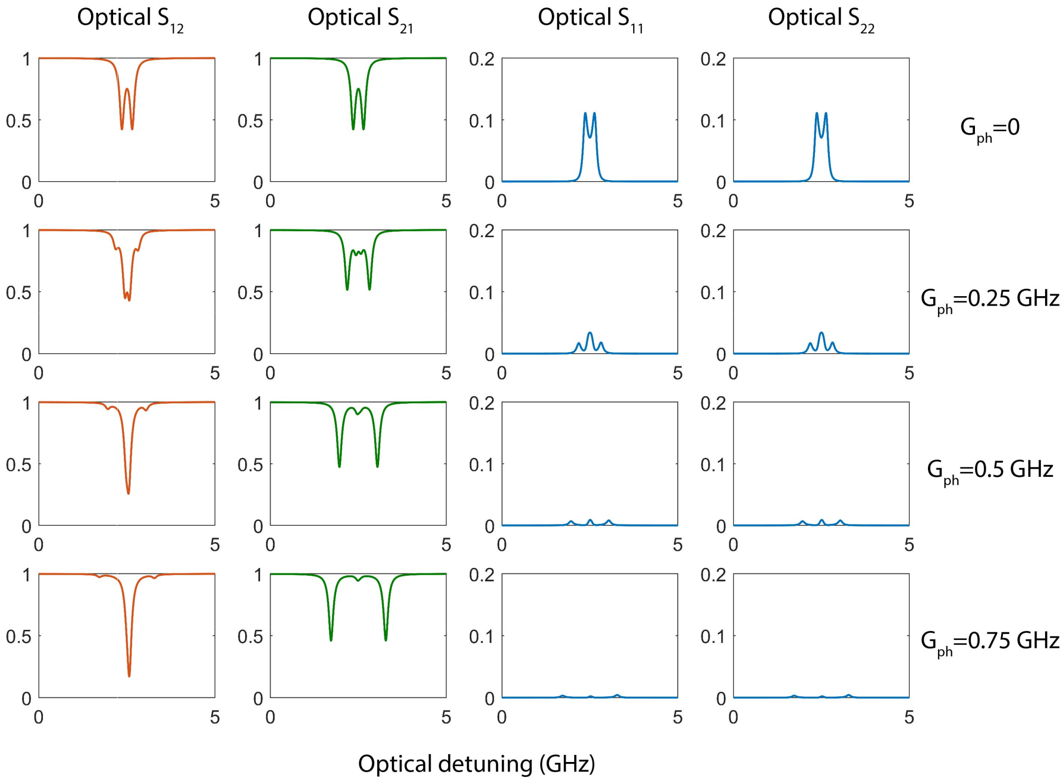

Figure S2: Example prediction of backscattering suppression and observation of optical S-parameters (reflection and transmission coefficients). Selected parameters are $\kappa_1 = \kappa_2 = 0.1$ GHz, $V_1/2 = V_2/2 = 0.15$ GHz, $\kappa_{ex1} = \kappa_{ex2} = 0.05$ GHz, $\omega_1 - \omega_2 = 5$ GHz and $\Omega = 5$ GHz. We show the evolution of the optical S-parameters under increasing acousto-optic coupling rate G_{ph}.

S4 Transformation of the frequency basis for equivalent system representation

The two-level photonic system with backscattering can be transformed into a coupled resonator chain with 4-sites. To show this equivalence, we start with the equations of motions in the original static frame where we neglect any acousto-

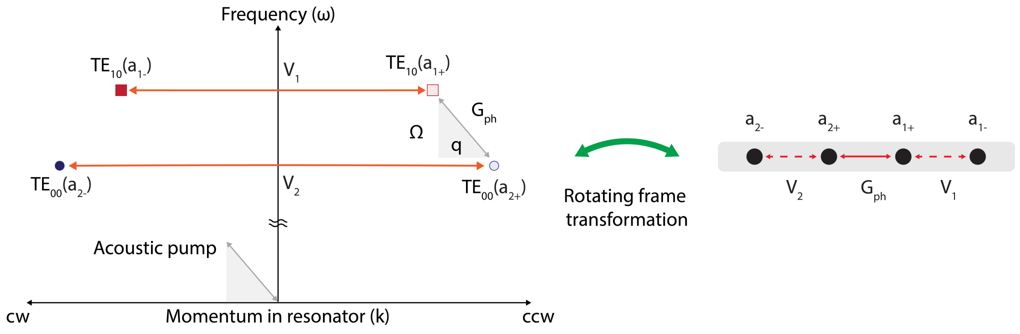

Figure S3: Transformation of a two-level photonic system to a 1D coupled resonator chain. The forward and backward optical modes of a two-level photonic system have distinct frequencies and momentum, and they cannot be represented with a static Hamiltonian (i.e., without oscillating terms). However, with appropriate choice of a rotating frame as $e^{i\Omega t}$, the two-level photonic system can be described by a 1D coupled resonator chain.

optic interaction in the non-phase matched direction:

$$\frac{d}{dt}\begin{pmatrix} a_{1+} \\ a_{2+} \\ a_{1-} \\ a_{2-} \end{pmatrix} = -i \begin{pmatrix} \omega_1 - i\frac{\kappa_1}{2} & \frac{G_{ph}}{2}e^{-i\Omega t} & \frac{V_1}{2} & 0 \\ \frac{G_{ph}}{2}e^{i\Omega t} & \omega_2 - i\frac{\kappa_2}{2} & 0 & \frac{V_2}{2} \\ \frac{V_1}{2} & 0 & \omega_1 - i\frac{\kappa_1}{2} & 0 \\ 0 & \frac{V_2}{2} & 0 & \omega_2 - i\frac{\kappa_2}{2} \end{pmatrix} \begin{pmatrix} a_{1+} \\ a_{2+} \\ a_{1-} \\ a_{2-} \end{pmatrix}. \tag{S30}$$

Then, we change the frame of reference, where we choose a frame in which modes are transformed as $a_{1\pm} \to a_{1\pm}$ and $a_{2\pm} \to a_{2\pm}e^{i\Omega t}$. As a consequence, each equation of motion transforms as:

$$\begin{aligned} \dot{a_{1+}} &= -i\left[\left(\omega_1 - i\frac{\kappa_1}{2}\right)a_{1+} + \frac{G_{ph}}{2}\cancel{e^{-i\Omega t}}(a_{2+}\cancel{e^{i\Omega t}}) + \frac{V_1}{2}a_{1-}\right] \\ &= -i\left[\left(\omega_1 - i\frac{\kappa_1}{2}\right)a_{1+} + \frac{G_{ph}}{2}a_{2+} + \frac{V_1}{2}a_{1-}\right] \end{aligned} \tag{S31}$$

$$\begin{aligned} \frac{d}{dt}\left(a_{2+}e^{i\Omega t}\right) &= \dot{a_{2+}}\cancel{e^{i\Omega t}} + i\Omega a_{2+}\cancel{e^{i\Omega t}} \\ &= -i\left[\frac{G_{ph}}{2}\cancel{e^{i\Omega t}}a_{1+} + \left(\omega_2 - i\frac{\kappa_2}{2}\right)a_{2+}\cancel{e^{i\Omega t}} + \frac{V_2}{2}a_{2-}\cancel{e^{i\Omega t}}\right] \\ \therefore \dot{a_{2+}} &= -i\left[\frac{G_{ph}}{2}a_{1+} + \left(\omega_2 + \Omega - i\frac{\kappa_2}{2}\right)a_{2+} + \frac{V_2}{2}a_{2-}\right] \end{aligned} \tag{S32}$$

$$\dot{a_{1-}} = -i\left[\frac{V_1}{2}a_{1+} + \left(\omega_1 - i\frac{\kappa_1}{2}\right)a_{1-}\right] \tag{S33}$$

$$
\begin{aligned}
\frac{d}{dt}\left(a_{2-}e^{i\Omega t}\right) &= \dot{a_{2-}}\cancel{e^{i\Omega t}} + i\Omega a_{2-}\cancel{e^{i\Omega t}} \\
&= -i\left[\frac{V_2}{2}a_{2+}\cancel{e^{i\Omega t}} + \left(\omega_2 - i\frac{\kappa_2}{2}\right)a_{2-}\cancel{e^{i\Omega t}}\right] \\
\therefore a_{2-} &= -i\left[\frac{V_2}{2}a_{2+} + \left(\omega_2 + \Omega - i\frac{\kappa_2}{2}\right)a_{2-}\right]
\end{aligned}
\tag{S34}
$$

Therefore, in the chosen frame of reference, the equation of motion becomes:

$$
\frac{d}{dt}\begin{pmatrix} a_{1+} \\ a_{2+} \\ a_{1-} \\ a_{2-} \end{pmatrix} = -i\begin{pmatrix} \omega_1 - i\frac{\kappa_1}{2} & \frac{G_{ph}}{2} & \frac{V_1}{2} & 0 \\ \frac{G_{ph}}{2} & \omega_2 + \Omega - i\frac{\kappa_2}{2} & 0 & \frac{V_2}{2} \\ \frac{V_1}{2} & 0 & \omega_1 - i\frac{\kappa_1}{2} & 0 \\ 0 & \frac{V_2}{2} & 0 & \omega_2 + \Omega - i\frac{\kappa_2}{2} \end{pmatrix}\begin{pmatrix} a_{1+} \\ a_{2+} \\ a_{1-} \\ a_{2-} \end{pmatrix},
\tag{S35}
$$

where the off-diagonal terms, which describe the interaction between different modes, form the interaction Hamiltonian. From equation S35, we see that the equivalence that is described in Fig S3 holds.

S5 Similarities between the photonic molecule and the SSH model

As described in the main text, the suppression of backscattering that we have demonstrated in this work can also be understood in terms of the topological phases of the well-known Su-Schrieffer-Heeger (SSH) model. We make this connection by interpreting the interaction Hamiltonian for the system as a short 1D SSH chain in a rotating reference frame (see Fig. S3 and §S4). In this model, the backscattering rates (V_1 and V_2) and the acousto-optical coupling rate (G_{ph}) can be interpreted as intra- and inter-unit-cell couplings, respectively, where control over G_{ph} allows us to adjust the topological phase of the system.

For example, when the system is in a weak acousto-optical coupling regime ($G_{ph} < V_1$, V_2), it can be considered equivalent to the topologically trivial phase of an SSH chain in which the inter-cell bonds are weak. In this trivial phase we should only able to observe bulk modes of the SSH chain that are gapped due to the Rayleigh scattering, which in our experiments appear in the form of the scattering induced modal doublet.

On the other hand, in the strong acousto-optical coupling regime ($G_{ph} \gg V_1$, V_2), the equivalent SSH chain experiences a topological transition into non-trivial phase. For a finite chain set up in this non-trivial phase, the states at the chain terminations should become exponentially localized and spectrally isolated within the bandgap. These modes will thus not couple to the rest of the bulk, i.e., they are topologically protected. In our experiment, the chain terminations

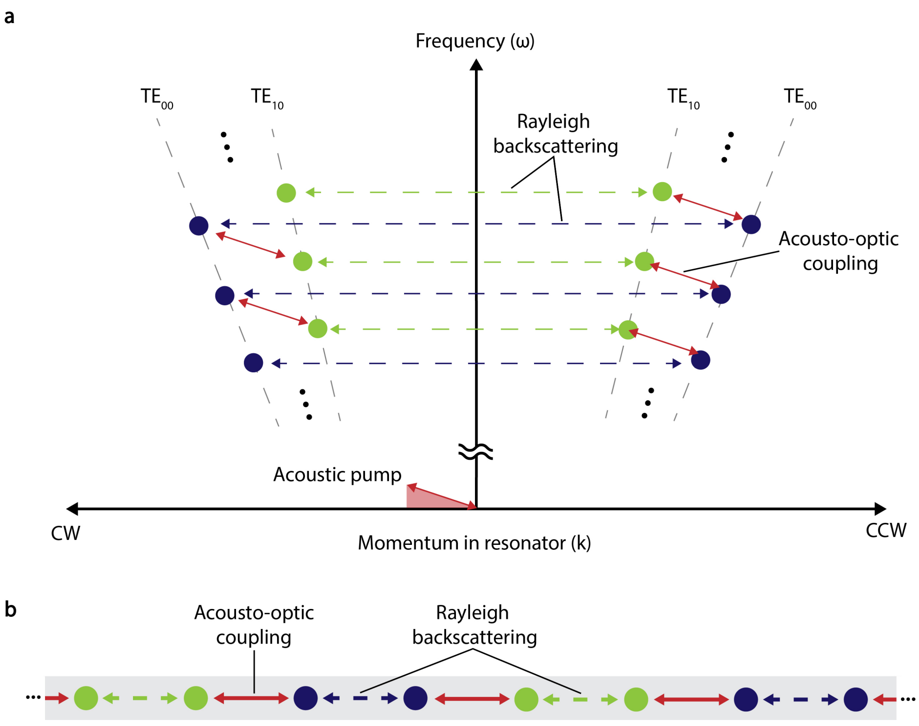

Figure S4: Extension of the system into longer SSH chains. (a) A frequency-momentum diagram proposing how Rayleigh backscattering and a single acousto-optic stimulus can couple multiple mode pairs into a 1-dimensional SSH chain. **(b)** Diagram of the equivalent SSH chain when considering rotating frame of reference. Chain terminations will eventually appear due to dispersive effects, i.e. when the phase matching between optical and acoustic modes is no longer satisfied.

correspond to the a_{1-} and a_{2-} modes (see Fig. S3), and we plainly observe this protection effect in the experiments since the a_{1-} and a_{2-} recover to their original Lorentzian shape when large G_{ph} is applied.

This observed topological protection of the a_{1-} and a_{2-} propagating modes can be lost if the phase matching is lost or if the acousto-optical coupling becomes weaker. The mechanism of the loss of topological protection in these two cases differs: in the former case, such as when the RF drive is mismatched ($\Omega \neq \omega_1 - \omega_2$) the chiral symmetry of the system is broken, and in the latter case, the system enters a topologically trivial phase.

While in our experiments the equivalent SSH chain is composed of only 2 unit cells, this chain could be readily be extended (Fig. S4) by imagining a larger racetrack resonator where multiple mode pairs are coupled simultaneously by a single acousto-optic stimulus. Having a larger racetrack resonator is helpful in this context for bringing the free spectral ranges (FSR) of the modes into a practical range for the acousto-optic stimulus Ω.

Optical dispersion engineering can be used to ensure that the two mode

families have similar dispersion, so this chain can in principle be quite long. Ultimately, as mentioned above, the slight difference in dispersion will ultimately produce a termination of the chain at some point in the spectrum.

Indeed, this system may also be extended to study higher-order topological structures by incorporating additional modes and acousto-optic stimuli into the system.

S6 Measurement of the Optical Reflection and Transmission

In order to measure the Stokes, anti-Stokes, and carrier transmission, we developed an optical heterodyne detection system by using an acousto-optic frequency shifter (Fig. S5)

Light is generated via a tunable external cavity diode laser (New Focus model TLB-6728-P) and split with a 50:50 coupler to realize a simple interferometer with two optical arms. One of the arms probes the device under test while the other arm is used as a reference. An acousto-optic frequency shifter is used to offset the reference path by Ω_r = 100 MHz for heterodyne detection via a high-speed photodetector (PD). The directionality of the probing is controlled by an optical switch. Fiber polarization controllers (FPCs) are used to change the polarization of the light that is coupled to the chip.

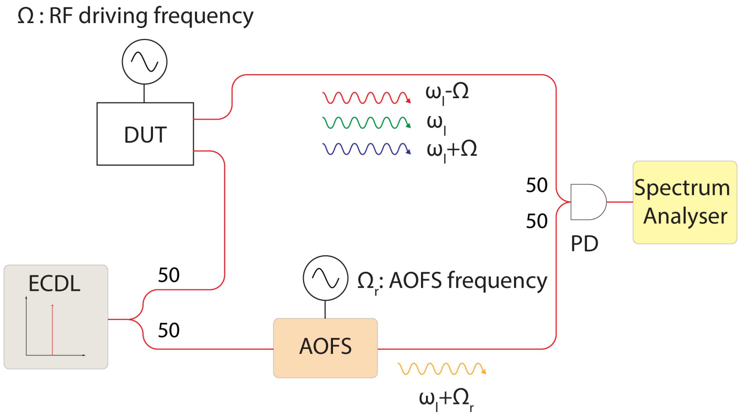

Figure S5: Heterodyne detection system. ECDL: External cavity diode laser, AOFS: Acousto-optic frequency shifter, PD: Photodetector, DUT: Device under test

We can write the optical spectrum arriving at the photo-detector as

$$s_{out} = s_{out,0}e^{i\omega_l t} + s_{out,-1}e^{i(\omega_l-\Omega)t} + s_{out,+1}e^{i(\omega_l+\Omega)t} + s_{out,r}e^{i(\omega_l+\Omega_r)t} \quad \text{(S36)}$$

The resulting RF photocurrent output has beat notes between the optical reference signal with the transmitted components at carrier, Stokes, and anti-Stokes frequencies, which appear at Ω_r, $\Omega+\Omega_r$ and $\Omega-\Omega_r$ respectively. Using equation S36, we can find the RF outputs from the photodetector as

$$P_{\Omega_r} = g_{pd}|s_{out,r}|^2|s_{out,0}|^2 \tag{S37}$$

$$P_{\Omega+\Omega_r} = g_{pd}|s_{out,r}|^2|s_{out,-1}|^2 \tag{S38}$$

$$P_{\Omega-\Omega_r} = g_{pd}|s_{out,r}|^2|s_{out,+1}|^2 \tag{S39}$$

Here P_{Ω_r}, $P_{\Omega+\Omega_r}$ and $P_{\Omega-\Omega_r}$ correspond to the RF power of each beat note in the photocurrent. We have used g_{pd} to represent the optical power to RF power conversion efficiency including photodetector gain. The input carrier power to the system is also measured by the photodetector, and is given by

$$P_{in} = g_{pd}|s_{out,r}|^2|s_{in}|^2 \tag{S40}$$

We can then normalize all the measured signals S37-S39 with respect to input power to obtain

$$\overline{P}_{\Omega_r} = |s_{out,0}/s_{in}|^2 \tag{S41}$$

$$\overline{P}_{\Omega+\Omega_r} = |s_{out,-1}/s_{in}|^2 \tag{S42}$$

$$\overline{P}_{\Omega-\Omega_r} = |s_{out,+1}/s_{in}|^2 \tag{S43}$$

To measure the optical back-reflection alongside the transmission, we modify our experimental setup as shown in Fig. S6. For convenience we set up field transfer coefficients for the waveguide couplers using variables η_1, η_2 and η_3, based on the power splitting ratios $\eta_1^2 = 0.9$, $\eta_2^2 = 0.1$, and $\eta_3^2 = 0.5$, and the laser output field is represented with E. At photodetector 1 (PD_1), we observe

$$s_{1,out} = ER_0\eta_1^2 e^{i\omega_l t} + ER_{-1}\eta_1^2 e^{i(\omega_l-\Omega)t} + ER_{+1}\eta_1^2 e^{i(\omega_l+\Omega)t} + E\eta_2^2\eta_3 e^{i(\omega_l+\Omega_r)t} \tag{S44}$$

Here, R_{i} represents the reflection coefficient for the corresponding frequency component (i). Similarly, we can write the output spectrum at photodetector 2 (PD_2) as

$$s_{2,out} = ET_0\eta_1^2 e^{i\omega_l t} + ET_{-1}\eta_1^2 e^{i(\omega_l-\Omega)t} + ET_{+1}\eta_1^2 e^{i(\omega_l+\Omega)t} + E\eta_2^2\eta_3 e^{i(\omega_l+\Omega_r)t} \tag{S45}$$

Here, T_{i} represents the transmission coefficient. Again, the resulting photocurrents are the beat notes of the optical reference with the carrier, Stokes, and anti-Stokes signals, and appear at Ω_r, $\Omega+\Omega_r$ and $\Omega-\Omega_r$, respectively. From equations S44 and S45, we can find the RF powers due to the photocurrents at both detectors as

$$P_{1,\Omega_r} = g_{pd}|E^2\eta_1^2\eta_2^2\eta_3 R_0|^2 \tag{S46}$$

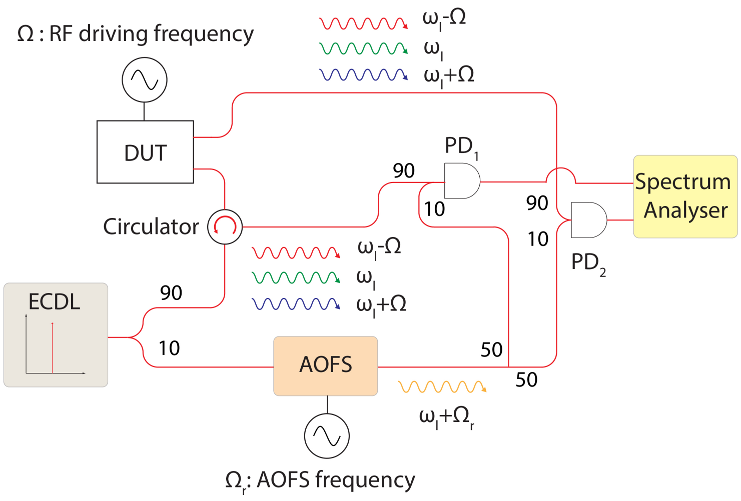

Figure S6: Measurement setup that simultaneously measures the reflection and the transmission coefficients. The light from the ECDL is split into two reference arms with a single probe arm that has a circulator. Both reflected and transmitted signals are measured by the beat note signals that are obtained by the frequency-shifted AOFS signal.

$$P_{1,\Omega+\Omega_r} = g_{pd}|E^2\eta_1^2\eta_2^2\eta_3 R_{-1}|^2 \tag{S47}$$

$$P_{1,\Omega-\Omega_r} = g_{pd}|E^2\eta_1^2\eta_2^2\eta_3 R_{+1}|^2 \tag{S48}$$

$$P_{2,\Omega_r} = g_{pd}|E^2\eta_1^2\eta_2^2\eta_3 T_0|^2 \tag{S49}$$

$$P_{2,\Omega+\Omega_r} = g_{pd}|E^2\eta_1^2\eta_2^2\eta_3 T_{-1}|^2 \tag{S50}$$

$$P_{2,\Omega-\Omega_r} = g_{pd}|E^2\eta_1^2\eta_2^2\eta_3 T_{+1}|^2 \tag{S51}$$

Where P_{1,Ω_r} (P_{2,Ω_r}), $P_{1,\Omega+\Omega_r}$ ($P_{2,\Omega+\Omega_r}$), and $P_{1,\Omega-\Omega_r}$ ($P_{2,\Omega-\Omega_r}$) are the carrier, Stokes and anti-Stokes RF powers representing the reflection (transmission) signals.

As before, the input carrier power is also measured by the photodetector 2 (PD$_2$), and is given by

$$P_{in} = g_{pd}|E^2\eta_1^2\eta_2^2\eta_3 s_{in}|^2 \tag{S52}$$

We can then normalize all the measured signals S46-S51 with respect to input power, and get

$$\overline{P}_{1,\Omega_r} = |R_0/s_{in}|^2 \tag{S53}$$

$$\overline{P}_{1,\Omega+\Omega_r} = |R_{-1}/s_{in}|^2 \tag{S54}$$

$$\overline{P}_{1,\Omega-\Omega_r} = |R_{+1}/s_{in}|^2 \tag{S55}$$

$$\overline{P}_{2,\Omega_r} = |T_0/s_{in}|^2 \tag{S56}$$

$$\overline{P}_{2,\Omega+\Omega_r} = |T_{-1}/s_{in}|^2 \tag{S57}$$

$$\overline{P}_{2,\Omega-\Omega_r} = |T_{+1}/s_{in}|^2 \tag{S58}$$

We then use these normalized signals S53-S58 to fit our data with our model.

S7 RF Electrode Characterization

We fabricated aluminum interdigital transducers (IDTs) to excite the required surface acoustic waves (SAW) in X-cut thin film $LiNbO_3$. The cross-section of our material stack is given in Fig. S7a, which is composed of 500 nm $LiNbO_3$, 2 um SiO_3 and 500 um Si handle. Following our finite element simulations, we design and align our actuators along Y-30° to efficiently excite symmetric SAW modes in this stack. For experimental characterization, we use a calibrated network analyzer to measure the S_{11} parameters of the IDT. The experimental results are given in Fig. S7b. The reflection dip around 3 GHz corresponds to our the symmetric SAW mode corresponding to the electrode pitch and experimentally shows a very large electromechanical coupling rate. We use this acoustic mode to induce strong chiral dispersion in our micro-resonators.

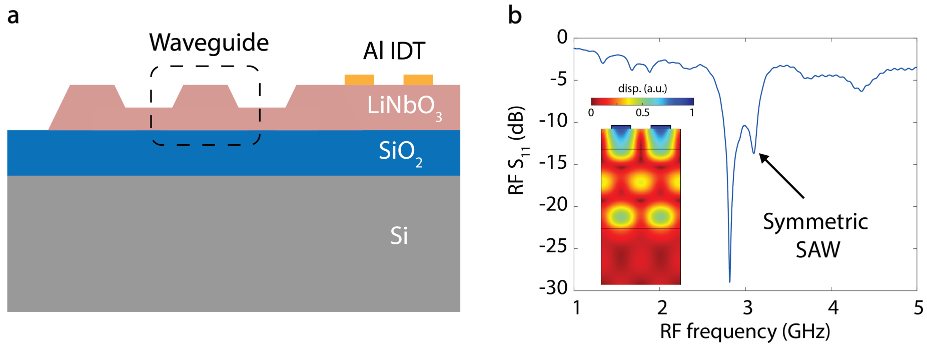

Figure S7: Characterization of the RF electrode and the surface acoustic wave (SAW) coupling efficiency. **(a)** Schematic of the device cross-section. **(b)** The IDT is characterized using RF reflection measurement (RF S_{11} parameter). Here we see that the RF reflection drops significantly around 3 GHz, indicating that the input RF power is efficiently coupled into the acoustic wave. This matches our finite element simulations for the symmetric SAW.

S8 Summary of experimental parameters

Table S1: Parameters of our experimental demonstration of backscattering suppression

	Parameters	Unit	Device in Fig. 3a	Device in Fig. 3b	Device in Fig. 4
Two-mode optical WGR	$\Delta\omega$ of TE_{10} and TE_{00} modes (ω_1 - ω_2)	GHz	3.06	3.06	3.18
	Total loss rate of TE_{00} mode (κ_2)	GHz	0.09	0.1	0.12
	Total loss rate of TE_{10} mode (κ_1)	GHz	1.01	1.01	1.08
	Quality factor of TE_{00} mode (Q_2)	-	2.15×10^6	1.93×10^6	1.76×10^6
	Quality factor of TE_{10} mode (Q_1)	-	1.92×10^5	1.92×10^5	1.79×10^5
	External coupling rate (κ_{ex2})	GHz	0.02	0.01	0.01
	External coupling rate (κ_{ex1})	GHz	0.59	0.68	0.88
	Rayleigh scattering within TE_{00} mode (V_2)	GHz	≈ 0	0.16	0.17
	Rayleigh scattering within TE_{10} mode (V_1)	GHz	≈ 0	0.21	0.27
	Wave number difference ($\lvert k_1 - k_2 \rvert$)	m^{-1}	2.47×10^5	2.47×10^5	2.47×10^5
	Center Wavelength	nm	1526	1524	1556
Surface Acoustic Wave	Transverse wave number ($q_{transverse}$)	m^{-1}	2.84×10^6	2.84×10^6	2.84×10^6
	Propagating wave number ($q_{propagating}$)	m^{-1}	2.47×10^5	2.47×10^5	2.47×10^5
	Total wave number (q_{total})	m^{-1}	2.85×10^6	2.85×10^6	2.85×10^6
	IDT Pitch (λ)	μm	2.2	2.2	2.2
	IDT Aperture (W)	μm	400	400	400
	IDT Angle (θ)	degree	4.98	4.98	4.98
	Center frequency (Ω)	GHz	3.06	3.06	3.02
Acousto-optic Interaction	Phonon-enhanced optomechanical coupling (at 27 dBm applied RF power)	GHz	0.68	0.66	0.87

References

[S1] Shao, L. *et al.* Microwave-to-optical conversion using lithium niobate thin-film acoustic resonators. *Optica* **6**, 1498–1505 (2019). URL https://opg.optica.org/optica/abstract.cfm?URI=optica-6-12-1498.

[S2] Orsel, O. E. *High quality integrated LiNbO3 acousto-optic platform for visible light spectrum*. Thesis, University of Illinois at Urbana-Champaign (2021). URL https://hdl.handle.net/2142/110858.

[S3] Agarwal, G. S. & Jha, S. S. Multimode phonon cooling via three-wave parametric interactions with optical fields. *Phys. Rev. A* **88**, 013815 (2013). URL https://journals.aps.org/pra/abstract/10.1103/PhysRevA.88.013815.

[S4] Bochmann, J., Vainsencher, A., Awschalom, D. D. & Cleland, A. N. Nanomechanical coupling between microwave and optical photons. *Nature Physics* **9**, 712–716 (2013). URL https://www.nature.com/articles/nphys2748.

[S5] Kim, S., Taylor, J. M. & Bahl, G. Dynamic suppression of Rayleigh backscattering in dielectric resonators. *Optica* **6**, 1016–1022 (2019). URL https://opg.optica.org/optica/abstract.cfm?uri=optica-6-8-1016.

[S6] Sohn, D. B., Örsel, O. E. & Bahl, G. Electrically driven optical isolation through phonon-mediated photonic Autler–Townes splitting. *Nature Photonics* **15**, 822–827 (2021). URL https://www.nature.com/articles/s41566-021-00884-x.

[S7] Zhang, Y. B., Darmawan, S., Mei, T. & Zhang, D. H. Temporal coupled mode theory in ring-bus-ring configuration. In *Advances in Optoelectronics and Micro/nano-optics*, 1–4 (2010). URL https://ieeexplore.ieee.org/document/5713512.
